# SecurePose: Automated face blurring and human movement kinematics extraction from videos recorded in clinical settings

**DOI:** 10.1038/s41598-026-50340-5

**Published:** 2026-05-02

**Authors:** Rishabh Bajpai, Bhooma Aravamuthan

**Affiliations:** Division of Pediatric Neurology, Department of Neurology, Washington University School of Medicine, 660 South Euclid Avenue, Campus Box 8111, St. Louis, MO 63110-1093 USA

**Keywords:** Face blurring, Patient data anonymization, Automation, Kinematics extraction, Software development

## Abstract

Movement disorder diagnosis often relies on expert evaluation of patient videos, but sharing these videos poses privacy risks. Current methods for de-identifying videos, such as blurring faces, are often manual, inconsistent, or inaccurate. Furthermore, these methods can compromise automated kinematics extraction—a valuable aid to movement analysis. To address these challenges, we developed SecurePose, open-source software that provides both reliable de-identification and automated body kinematic extraction from videos recorded in clinic settings using smartphones/tablets. We validated SecurePose on gait videos recorded in outpatient clinic visits of 116 children with cerebral palsy, assessing both the accuracy of its de-identification compared to the ground truth (manual blurring) and the reliability of the intermediate steps of kinematics extraction. Results demonstrate that SecurePose outperformed six existing methods in automated face detection and achieved comparable accuracy to robust manual blurring, but in significantly less time (91.08% faster). Ten experienced researchers also confirmed SecurePose’s usability via System Usability Scale scores. These findings validate SecurePose as a practical and effective tool for protecting patient privacy while enabling accurate kinematics extraction in clinical settings.

## Introduction

The potential for dramatically improving clinical care and accelerating medical research hinges on the broad sharing of medical data. However, realizing this potential is currently hampered by significant challenges in protecting patient privacy while simultaneously extracting clinically valuable information. A particularly rich data source is video recordings of clinical visits, crucial for diagnosing and tracking progression in numerous conditions, especially motor disorders^1–4^. These recordings are increasingly used for detailed, quantitative assessments unattainable through traditional methods, driven by advancements in pose estimation and kinematic analysis^5^. However, responsibly sharing this data requires robust anonymization techniques, and existing methods are either prohibitively time consuming (i.e. manual blurring) or inaccurate, risking patient privacy (i.e. automated face blurring).

Current regulations, such as the Health Insurance Portability and Accountability Act (HIPAA)^6^ in the United States, the General Data Protection Regulation (GDPR)^7^ in the European Union, and the Personal Data Protection Bill (PDPB)^8^ in India, all emphasize the need for stringent protection of personally identifiable information including facial images. Consequently, any attempt to broadly share video data must prioritize complete removal of identifying features, such as faces, before storage or distribution. While manual blurring is the current gold standard, it is a time consuming, labor-intensive, and error-prone process, hindering the large-scale data sharing necessary for robust research.

A spectrum of automated face detection methods have been proposed, each with its strengths and weaknesses. Early approaches relied on feature-based techniques, such as the Viola-Jones algorithm^9^ and Histogram of Oriented Gradients (HOG)^10^. These methods are computationally efficient and relatively straightforward to implement, making them suitable for real-time applications. However, they can struggle with blurring faces in images with variations in pose, lighting, and occlusion, potentially leading to missed or inaccurate face detections or blurring regions^9,10^.

More recently, image-based techniques leveraging convolutional neural networks (CNNs) have gained prominence. Algorithms like Faster R-CNN^11^, Single Shot MultiBox Detector (SSD)^12^, max-margin object detection (MMOD)^13^, single shot scale-invariant face detector (S^3^FD)^14^, and you only look once (YOLO)^15^ offer significantly improved accuracy and robustness compared to traditional feature-based methods^16,17^. These algorithms excel at detecting faces in complex scenes and under challenging conditions^16,17^. However, they require substantial computational resources and large training datasets, potentially limiting their practicality in resource-constrained settings^16,17^. Multi-task cascaded convolutional network (MTCNN)^18^ offers a good balance between performance and efficiency, becoming a popular choice for many applications. However, even these advanced algorithms can struggle with specific scenarios, particularly those involving low-resolution video, unusual angles, or obscured facial features^11–17^. Furthermore, many existing algorithms were designed for static images and may not maintain temporal consistency across video frames, resulting in flickering or inconsistent blurring.

Extracting body kinematics from video recordings, facilitated by tools like OpenPose^19^, is revolutionizing the assessment of motor disorders, moving beyond subjective evaluations to quantifiable, objective measures. However, performing pose estimation on videos that have already been anonymized poses a specific methodological challenge. When face blurred datasets are shared for secondary analysis, the blurring artifacts can negatively impact the accuracy of kinematic data extraction^20^. Specifically, performing pose estimation on face blurred videos can introduce error, as algorithms may erroneously interpret blurred regions as body parts or fail to track head positioning accurately. Therefore, to ensure data quality, it is crucial to perform kinematic data extraction on the raw footage before applying anonymization techniques.

To advance research in motor disorders, a dedicated pipeline optimized for clinical video data is crucial, prioritizing both accurate anonymization and reliable body kinematic analysis. In this study, we develop and validate SecurePose, open-source software capable of reliably identifying and obscuring patient faces while preserving the integrity of extracted body kinematic data, overcoming the limitations of existing methods.

## Methods

2.1. Dataset description.

We obtained ethical approval from the Washington University in St. Louis Institutional Review Board (approval number 202102101). Informed consent was obtained from all participants (and caregivers for minors) prior to their inclusion in the study and use of their clinical data, including video recordings. All methods were performed in accordance with the relevant guidelines and regulations. All identifying information, including subjects’ images and features, has been excluded from the manuscript to protect subject’s privacy. Participants were recruited from the St. Louis Children’s Hospital Cerebral Palsy Clinic between January 1, 2020, and November 4, 2021. A total of 180 individuals met the following inclusion criteria: age 10–20 years at the time of evaluation, diagnosis of cerebral palsy according to the 2006 consensus diagnostic criteria^21^, presence of spasticity, and independent ambulation (Gross Motor Function Classification System levels I-III)^22^. This age range was selected to capture a period of relative gait pattern maturity and gross motor function stabilization in children with cerebral palsy^23^. Following initial screening, 64 individuals were excluded due to the absence of gait videos demonstrating full body visualization, including the feet. Therefore, a final cohort of 116 participants, contributing 116 gait videos, met all inclusion and exclusion criteria and were included in the subsequent analysis.

2.2. Protocol and data collection.

To assess the practical applicability of SecurePose in clinical settings, standard gait data collected during clinic visits was used. Participants walked approximately 15 feet in a straight line along a clinic hallway towards a stationary camera. Videos were recorded using an Apple iPad A1432 at 30 fps and 640 × 360 pixels resolution. The iPad was handheld by clinic staff, introducing practical challenges commonly encountered in clinical video analysis, such as motion blur, variable focus, lighting changes, and video shaking. SecurePose was specifically developed and validated using this clinical dataset to address these real-world challenges.

### Overview of software design

**Fig. 1:**
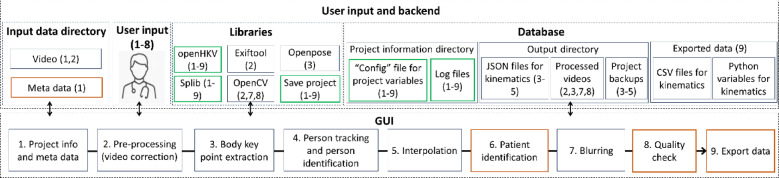
Logic flow diagram of SecurePose and pictorial representation of key intermediate steps used by SecurePose. The green-colored boundary box modules are called during the execution of each step. The orange-colored boundary box modules are optional steps. Each ‘GUI’ element represents each of 9 steps taken by SecurePose to perform blurring and kinematics extraction. ‘User input and backend’ elements are used by SecurePose for input, processing, and output during these 9 steps, as is indicated parenthetically. For example, ‘Video’ is used during the execution of GUI steps 1 and 2, which are ‘Project info and metadata’ and ‘Pre-processing (video correction)’.

Prior to clinical movement data processing, several essential steps are required, including video pre-processing (video correction), facial anonymization, and accurate extraction of movement kinematics. The logic flow diagram of SecurePose, used to automate these tasks, is presented in Fig. 1. SecurePose is implemented in Python, with a graphical user interface (GUI) developed using TKinter^24^. Hardware and software requirements for SecurePose are detailed in its SourceForge repository^25^. To organize the functions, classes, and variables, we developed two libraries: openHKV (open human kinematics vision) and splib (secure pose library).

### Project info and metadata

SecurePose initiates with a screen prompting users to enter project information and patient metadata. Users can either load a previously saved project or create a new project by providing a unique name and selecting the videos to be processed, as well as directories for output data. Optional patient metadata can also be associated with the videos. Any files containing information about the patient can be linked to the video in the SecurePose’s database as the video’s metadata. Examples of useful metadata include demographic data, clinic notes, and visit information. Once the metadata is linked, it can be shared along with the blurred video. The purpose of metadata linking is to better organize the project. These files should share the same base filename as the corresponding video file (e.g., “12,345.xlsx” for “12,345.mp4”) to enable automatic linking. Upon entering project information, a configuration file and log files are created within a dedicated project directory. This configuration file, created using the Python pickle library^26^, stores all project-related parameters and is loaded upon opening a previously created project. The software supports simultaneous processing of multiple video files for both kinematics extraction and face blurring.

### Pre-processing: video standardization

Videos recorded from different devices such as mobile phones, tablets and cameras can have different hardware biases and video properties. These inconsistencies in file format and video properties could be problematic for body kinematic and face detection algorithms. To solve this problem, SecurePose automatically scans and standardizes all the videos in the project to ensure shared video properties and removal of hardware biases. The video standardization done by SecurePose includes correction of video orientation (i.e., rotation is corrected so that the patient is vertical) and audio subtraction (i.e., allows the user to mute the video). Specifically, videos are standardized to the .mp4 file format using the mp4v video codec, while preserving their original resolution (width and height) and framerate (fps). To perform the video standardization, a custom script, and modules of the Exiftool and OpenCV libraries were used.

### Body key point extraction

**Fig. 2:**
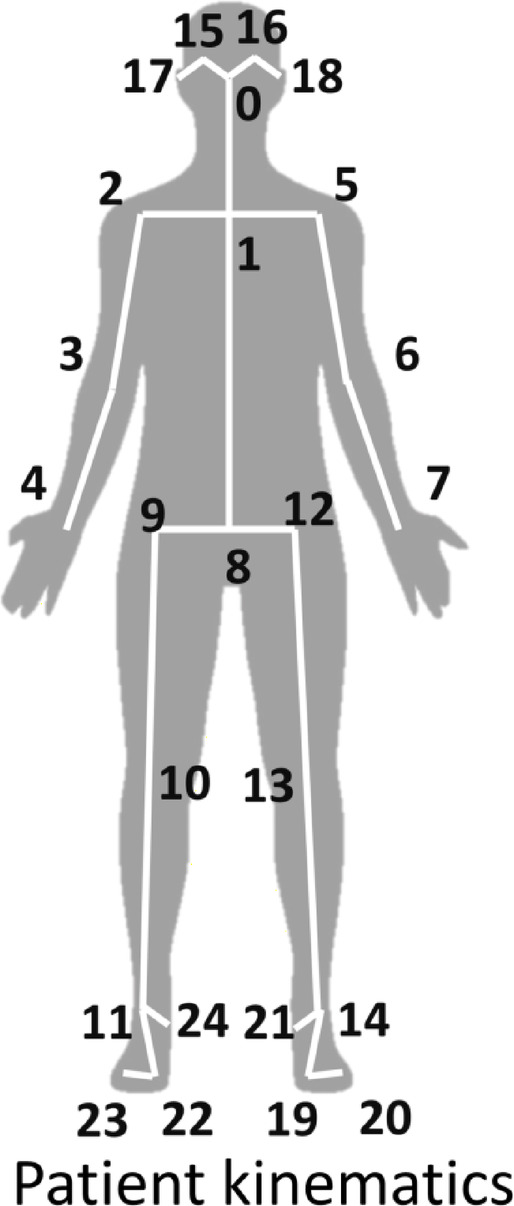
The index value and location of body key points with respect to the human body.

Following video pre-processing, we utilized the Windows implementation of OpenPose to extract the coordinates of 25 body keypoints (Fig. 2), as described in the original OpenPose publication^19^. OpenPose was selected for this work due to its high performance in human pose estimation and inclusion of clinically relevant body keypoints. At each video frame, OpenPose provides two-dimensional (2D) coordinates for each keypoint, along with a confidence level ranging from 0 to 1, representing the algorithm’s certainty regarding the accuracy of the keypoint position. We employed the “BODY 25” model of OpenPose due to its widely used and clinically relevant configuration, which has been demonstrated in OpenPose’s documentation to provide higher accuracy than the conventional common objects in context (COCO) and Max Planck Institute (MPI) models^27,28^, as well as its inclusion of foot keypoint labels, which are critical for our analysis. To avoid memory errors during keypoint extraction, we utilized the default resolution settings of OpenPose. The extracted body keypoint coordinates were stored separately for each frame in JavaScript Object Notation (JSON) format. The SecurePose GUI allows users to save the OpenPose output videos rendered with extracted body keypoints to a local directory and visualize the kinematics extraction in real-time.

### Person tracking and person identification

**Fig. 3:**
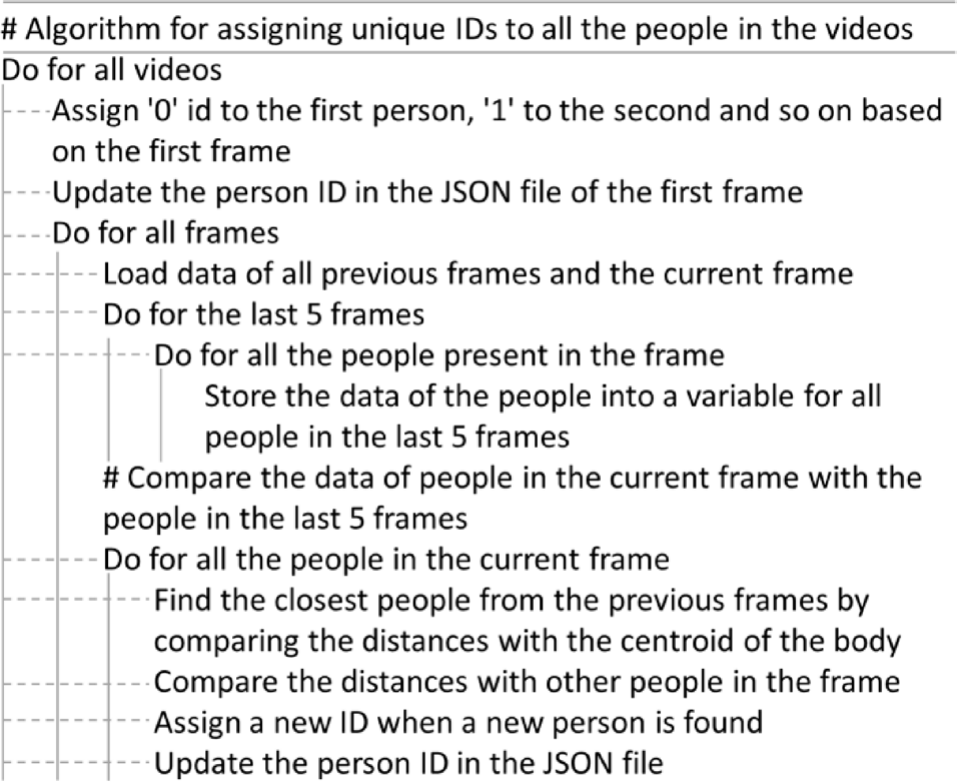
Algorithm for assigning unique IDs to all the people in the videos of a project.

OpenPose extracts body keypoints from videos frame-by-frame, but does not track these points across frames. To enable tracking, a custom Python algorithm was developed to identify individuals and assign them unique IDs based on their movement (Fig. 3). The algorithm iterates through video frames (JSON files), tracking the centroid of each OpenPose-detected person. It assigns unique IDs to new individuals while maintaining existing IDs for those already in the frame. Tracking is achieved by calculating the distance between a person’s current centroid and their averaged centroid from the previous five frames. The choice of the number of frames in the time window can significantly affect person tracking. Selecting a small window size increases susceptibility to pose estimation errors due to limited temporal context, while choosing a large window may smooth out rapid movements, thereby reducing the system’s ability to capture dynamic changes in motion. The algorithm then updates the JSON files by adding these unique IDs to the extracted keypoints, ensuring each person is uniquely identifiable throughout the video. This approach prioritizes reduced computation time and complexity, relying on the assumption that patient and staff centroids generally won’t collide in clinical recordings. Since averaging centroids across frames helps account for occasional OpenPose labeling errors, a 5-frame average was chosen to best represent movement given the 30fps video rate.

### Interpolation

**Fig. 4:**
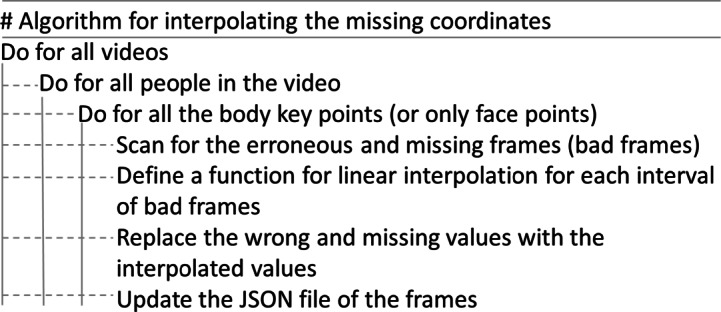
Algorithm for interpolating the missing coordinates of body key points.

Keypoint coordinates extracted by OpenPose are occasionally erroneous or undetected, particularly for visually occluded body parts. To address this, a custom algorithm was applied to the body keypoints identified for each participant (JSON files; see Fig. 4). Erroneous coordinates were defined a priori as those with an OpenPose confidence level below 0.50. In our dataset, only 0.29% of frames contained erroneous values for at least one body part. Linear interpolation was employed to address these missing coordinates, with separate linear functions fitted for each interval of missing data. This ensured complete keypoint data throughout the video, crucial for reliable face tracking and subsequent blurring. Within the GUI, users can select between interpolating only face coordinates or interpolating the coordinates of the whole body. Interpolating only face coordinates significantly reduces processing time, requiring just 20.8% of the time needed for whole-body interpolation. This option is suitable for users focused solely on facial blurring or who prefer to analyze non-interpolated body kinematics. Conversely, interpolating the entire body’s kinematics is more computationally intensive but appropriate for applications requiring linearly interpolated kinematics for further analysis (e.g. for detection of gait events using 2D kinematics data^29,30^). Additionally, SecurePose saves both interpolated and non-interpolated body kinematics of the entire body (including the face) in the output folder, allowing users to utilize raw body coordinates for their specific applications. To further enhance robustness and control over data reconstruction, we have introduced a user-configurable maximum interpolation gap parameter. This allows users to define the maximum number of consecutive frames for which interpolation is applied. When a gap exceeds this threshold, interpolation is skipped for that interval to avoid over-smoothing or the generation of unrealistic kinematic trajectories. This feature prevents the propagation of erroneous data in cases of prolonged occlusions or tracking failures and ensures that the output remains reliable for downstream analysis. The maximum interpolation gap is configurable via the GUI and is set to 10 frames by default.

While interpolation effectively addresses brief missing data, it is important to acknowledge its potential limitations. Interpolated data mathematically alters original motion dynamics and may suppress high-frequency kinematic variations or rapid gait events, potentially impacting the clinical validity of the derived metrics if extensive gaps are interpolated. To accommodate researchers who wish to avoid these alterations, SecurePose natively exports the uninterpolated raw body kinematics alongside the interpolated data, allowing users to choose the dataset that best fits their clinical analysis needs.

### Patient identification

While tracking all individuals within a video is valuable, for clinical purposes, accurate patient identification is paramount. Several automated approaches are possible, including deep learning, activity-specific movement recognition, and rule-based methods to identify the primary subject. However, deep learning and activity-specific movement recognition require extensive training datasets and may falter in clinically relevant scenarios, such as when patients exhibit minimal movement abnormalities or when non-patient individuals perform similar activities (like walking with the patient). Therefore, we adopted a method identifying the patient based on their prominence within the video recording. Initially, we tracked the movement of the body centroid for all individuals (each assigned a unique ID) throughout the video. Individuals present in fewer than 80% of frames were excluded, assuming clinically relevant videos capture the patient within the camera’s field of view for at least 80% of the duration. Subsequently, we computed the average Euclidean distance between each remaining individual’s body centroid and the center of the video frame across all frames. The algorithm identifies the individual with the smallest average Euclidean distance to the video frame center (closest to the center) as the primary subject (i.e., patient) in the clinical videos.

#### Blurring

Facial blurring was implemented using a Python script. The script initially reads facial coordinates derived from body key points extracted and stored in JSON files for each individual, separately. The centroid of these coordinates was then calculated using the median values across all facial coordinates in each frame in the X and Y directions to mitigate the impact of potential outliers. Given the potential for high-amplitude head movements in individuals with cerebral palsy, accurate estimation of facial dimensions presents a challenge. Furthermore, the presence of small faces necessitates minimizing estimation errors to ensure effective anonymization. Therefore, facial dimensions were estimated based on the length of the spine – defined as the distance between the neck and pelvis key points – leveraging established human body proportions. Specifically, the facial length and width were assumed to be equal to one-third of the spine length^31^. The GUI provides users with options for person-specific blurring (patient-only’ and 'all-people’) and allows selection of the desired blurring type (Gaussian or solid). While Gaussian-based blurring is commonly used and effective for obscuring facial identity, it is important to note that complete privacy cannot be guaranteed unless the entire face is obscured, as reconstruction risks may persist with partial blurring. In line with this, SecurePose offers users the option to apply solid blurring, which provides stronger privacy protection for those who prefer it over Gaussian blurring.

#### Quality check

Following automated blurring, the GUI presents an interactive window for quality assessment and refinement. Users can navigate through videos and individual frames to identify potential blurring errors. The GUI enables targeted correction, allowing users to either de-blur specific frames or manually delineate face regions for re-blurring. This interactive process ensures a high level of anonymization quality control.

#### Export data

A window in the menu bar is designed to selectively export the project data to an external directory. Users can export the blurred videos, project back-up files, and coordinates of body key points (as JSON, CSV, or python variables).

#### System specifications

For analysis, a system having the following specifications was used: IntelR Core™ i9-12900 CPU @ 2.40 GHz 16 cores, 32-GB RAM, NVIDIA GeForce RTX3080 GPU with 10 GB memory, 64-bit Windows 10 operating system, and MATLAB 2022b platform. Other hardware and system requirements are mentioned in the software documentation^25^.

#### Effect of face blurring on body key point extraction

To quantify the effect of face blurring on body keypoint extraction accuracy, we compared the coordinates extracted from blurred and pre-blurred videos. This analysis was conducted to assess potential issues that may arise when processing videos with pre-applied face blurring, such as those commonly found in pre-face blurred shared datasets, rather than to evaluate the performance of SecurePose itself. First, body keypoint coordinates were extracted from 116 pre-blurred videos using the SecurePose kinematics extraction pipeline. Subsequently, coordinates were extracted from manually face blurred videos (details of the manual blurring process are described in the following section) using the same pipeline. We considered the coordinates extracted from pre-blurred videos as the ground truth and calculated the error introduced by blurring as the Euclidean distance between corresponding keypoints. Additionally, we compared the confidence levels of keypoint estimation between pre-blurred and blurred videos. The results provide insight into how prior face blurring may affect downstream pose estimation, which is important for understanding the compatibility of SecurePose with anonymized datasets.

#### Comparison with six existing face blurring techniques

To evaluate the performance of SecurePose, we compared it with six widely used face detection methods using our clinically acquired video dataset. Table 1 details the trained models employed and the rationale for selecting each method. Since person-specific blurring was not feasible with the six existing methods, SecurePose was configured for “all-person blurring” for this comparative analysis (i.e. blurring the faces of all individuals present in the video).

**Table 1:** Selected existing methods for the comparison.

Model name	Original Article	Download link for trained model	Reason to select
Viola-Jones (VJ)	^9^	^32^	Fast face detector
HOG-based	^10^	^33^	Very low computational cost and more accurate than the VJ
MMOD	^13^	^34^	Outperformed other contemporary methods on the Face Detection Data Set and Benchmark (FDDB) challenge
MTCNN	^18^	^35^	Outperformed other contemporary methods on FDDB and WIDER Face, but computationally expensive
YOLO-face	^36^	^37^	Outperformed contemporary methods on FDDB and WIDER Face. One of the fastest methods
S^3^FD	^14^	^38^	Outperformed contemporary methods on Annotated Faces in the Wild (AFW), PASCAL face, FDDB and WIDER Face

All methods were compared against a manual face blurring standard: 116 videos of individuals with cerebral palsy walking (dataset described above) were manually blurred by an experienced clinical researcher using Shotcut, an open-source video editing software^39^. The veracity of this manual blurring was independently confirmed by another experienced clinical researcher through frame-by-frame review. For the manual blurring process, a standardized annotation protocol was followed where the bounding boxes were tightly aligned to the visible facial boundaries (in accordance with standard face benchmark guidelines such as WIDER FACE^40^, rather than being intentionally enlarged, to provide a precise ground truth for evaluation.

We assessed the face detection performance of all seven automated methods (SecurePose and the six selected existing methods) against this manual blurring (ground truth) using the 116-video dataset, encompassing a total of 19,753 frames. Intersection over Union (IoU) was calculated for all detected faces and the ground truth using Eq. 1.

1 $$IoU= \frac{{A}_{d} \cap {A}_{m}}{{A}_{d} \bigcup {A}_{m}}$$

where A_m_ represents the region of the image marked as a face by the manual blurring, and A_d_ represents the region detected as a face by the algorithm. A detection was considered correct if the Intersection over Union (IoU) value was greater than or equal to 0.5. This threshold corresponds to a detection overlapping at least half of the ground truth face region, which we selected as a standard benchmark to ensure meaningful spatial alignment while avoiding overly lenient criteria that could inflate false positives.

Automated methods were evaluated for:

1. True Positives (TP): A correct detection of ground truth face region. (IoU >  = 0.5 for ground truth face region)
2. False Positives (FP): An incorrect or a misplaced detection of a face region. (IoU < 0.5 for detected face regions)
3. False Negatives (FN): An undetected face region. (IoU < 0.5 for ground truth face regions)

In the context of face detection, the concept of true negatives (TN) does not apply. This is because there is an infinite number of regions representing areas without faces within any given frame^41^. The threshold determines how many of these detections are classified as TP, FP, or FN. At a lower IoU threshold (e.g., 0.3), more detections meet the criterion for TP, increasing recall and precision. Conversely, at a higher threshold (e.g., 0.75), only the most accurate detections qualify as TP, reducing recall and precision.

TP, FP, and FN were used to determine:

1) Precision: Precision is the ability of a model, when it detects a face, to have only detected ground truth face regions. A high precision value indicates that the model is able to accurately identify faces in an image without falsely detecting non-faces (see Eq. (2))

2 $$Precision= \frac{TP}{TP+FP}= \frac{TP}{All detections}$$

2) Recall: Recall is the ability of a model to detect all ground truth face regions. A high recall value indicates that the model is able to detect most of the faces in an image (see Eq. (3)).

3 $$Recall= \frac{TP}{TP+FN}= \frac{TP}{All ground truths}$$

3) F1-score: F1-score can be interpreted as a measure of overall model performance from 0 to 1, where 1 is the best. The F1-score can be interpreted as the model’s balanced ability to both capture positive cases (recall) and be accurate with the cases it does capture (precision) (see Eq. (4)).

4 $$F1-score= \frac{(2 * Precision * Recall)}{(Precision+Recall)}$$

4) Precision-recall curve: Precision-recall characteristics were calculated as per the method used in PASCAL VOC 2012 challenge^41^. However, both the Viola–Jones (VJ) and MMOD methods do not provide confidence scores for their detections. Since confidence scores are required to perform precision–recall analysis, it was not possible to generate precision–recall curves for these methods.

5) Average precision (AP): As described in the PASCAL VOC 2012 challenge^41^, 11-point interpolation was used to calculate AP.

#### Comparison of two pose estimation algorithms

OpenPose was selected as our primary pose estimation algorithm due to its high performance in human pose estimation and its inclusion of clinically relevant body keypoints, which are essential for our application. However, OpenPose demands significant computational resources, such as high VRAM and a modern GPU, for fast processing of high-definition videos, which may limit the software’s usability in resource-constrained clinical environments. In contrast, MediaPipe offers reliable human pose estimation with substantially lower computational requirements, making it a promising alternative for deployment in such settings. To evaluate the trade-offs between accuracy and efficiency, we conducted a comprehensive comparison between OpenPose and MediaPipe, assessing their performance across key metrics including precision, recall, F1-score, and average precision and time taken for automatic blurring of all videos.

#### System Usability Scale (SUS)

To determine the usability of SecurePose in clinical and research settings, ten clinical researchers underwent a training session on how to use the software (∼10 min), were then asked to use the software, and finally asked to give feedback using the System Usability Scale (SUS)^42^. SUS scores range from 0 to 100, with a reported average of 68 across published literature. A score exceeding 80.3 indicates software usability within the top 10 percentile^43^. The participating clinical researchers represented diverse professional backgrounds (five with expertise in gait and clinical video analyses, three with experience in clinical video analysis, and two with no prior experience in gait or clinical video analysis), allowing for a comprehensive assessment of software usability across varying levels of expertise.

## Results

### Effect of face blurring on body key point extraction

To evaluate the effect of blurring on body keypoint coordinate extraction, two metrics were considered: 1) confidence level (generated for each x, y coordinate pair by OpenPose), and 2) error (Euclidean distance between coordinates for each body keypoint generated by OpenPose pre- and post-face blurring).

Table 2 presents the mean confidence level for each key body point in pre-blurred and blurred videos across all frames, along with the mean coordinate error extracted from the face blurred videos. Results indicate a significant decrease in confidence levels for all body keypoints following blurring (p < 0.05, one-way ANOVA), with particularly low values (< 0.75) observed for points adjacent to the blurred face (shoulders and chest). Importantly, coordinate differences were observed for all body keypoints between pre- and post-blurred videos, demonstrating that blurring introduced errors in keypoint extraction.

**Table 2:** Mean confidence level for coordinates extraction in pre-blurred and blurred videos, and mean absolute error for coordinates extraction in blurred videos.

Body keypoint (Fig. 2)	Confidence level (pre-blurred)	Confidence level (blurred)	Error (pixel)
0–mid head	0.868	0.315	14
1–upper chest	0.890	0.692	8
2–right shoulder	0.891	0.708	7
3–right elbow	0.920	0.905	4
4–right wrist	0.940	0.934	3
5–left shoulder	0.893	0.710	8
6–left elbow	0.908	0.895	4
7–left wrist	0.899	0.892	3
8–mid pelvis	0.948	0.934	3
9–right pelvis	0.887	0.866	3
10–right knee	0.930	0.910	4
11–right lateral ankle	0.866	0.844	4
12–left pelvis	0.852	0.840	5
13–left knee	0.891	0.889	4
14–left lateral ankle	0.886	0.872	4
15–right eye	0.861	0.309	16
16–left eye	0.881	0.310	15
17–right ear	0.847	0.250	16
18–left ear	0.848	0.211	17
19–left medial toe	0.882	0.764	4
20–left lateral toe	0.859	0.803	4
21–left medial ankle	0.846	0.769	5
22–right medial toe	0.856	0.791	5
23–right lateral toe	0.849	0.834	3
24–right medial ankle	0.834	0.829	4

While OpenPose is not designed to track individuals across video frames, it should accurately differentiate between individuals and their associated keypoint coordinates within a single frame. However, instances were observed where OpenPose associated keypoint coordinates with the incorrect individual in blurred frames. Furthermore, blurring led to instances of OpenPose incorrectly identifying the laterality of body parts (left vs. right). These findings underscore the importance of performing coordinate extraction before applying face blurring to ensure accurate readings.

### Automatic face detection

**Fig. 5:**
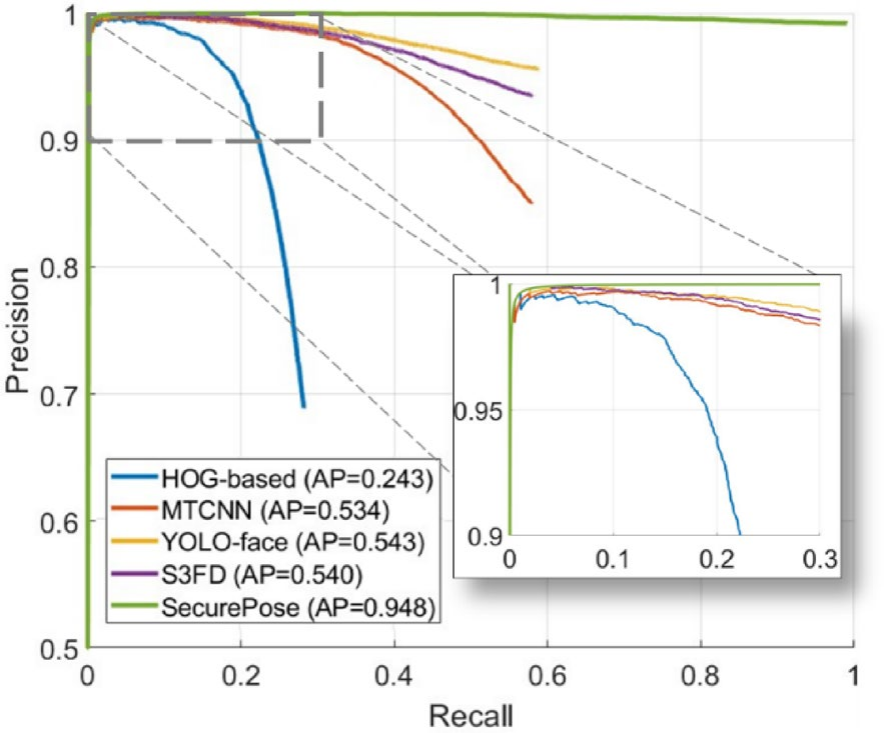
Precision-recall curve with average precision values for SecurePose and selected existing methods.

**Fig. 6:**
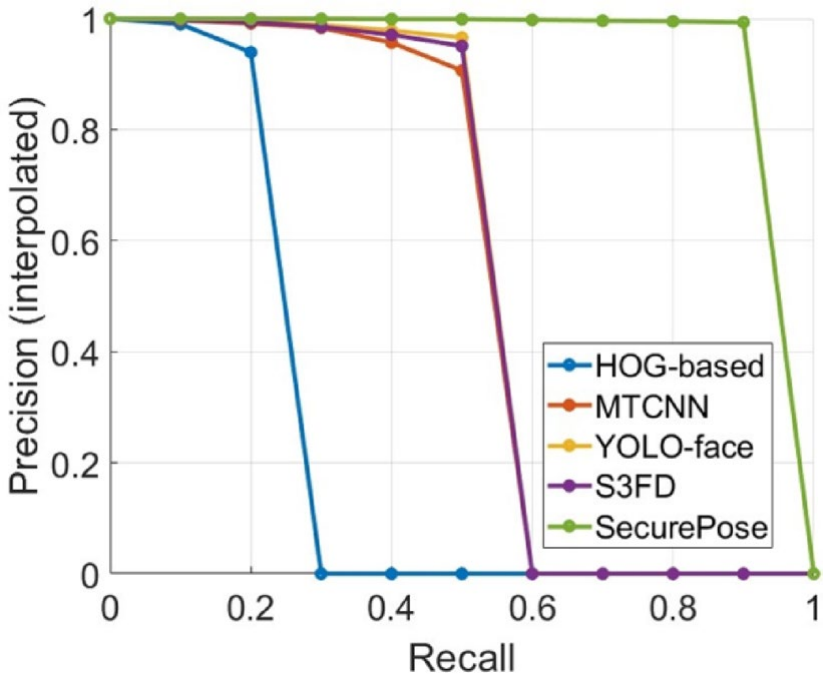
Eleven points interpolated precision-recall curve for SecurePose and selected existing methods.

The performance of each method was evaluated and is presented in Table 3, Figs. 5, and 6. SecurePose achieved high precision (0.992) and recall (0.990), yielding a robust F1-score of 0.991, demonstrating its proficiency in accurately and reliably detecting faces within clinical video data. As shown in Table 3, SecurePose and three of the six compared methods achieved precision values exceeding 0.9, indicating a high likelihood of correct face detection when a face was identified. The lowest precision was observed with the HOG-based detector (0.689). While some existing methods demonstrated comparable precision to SecurePose, none matched its high recall performance. Specifically, the six compared methods exhibited limited ability to detect all ground truth face regions, with recall values ranging from 0.282 to 0.588.

**Table 3:** Precision, recall, f1-score and average precision for the six selected existing methods and securepose.

Methods	Precision	Recall	F1-score	Average precision
Viola Jones	0.937	0.488	0.642	-
HOG-based detector	0.689	0.282	0.400	0.243
MMOD	0.822	0.376	0.516	-
MTCNN	0.850	0.579	0.689	0.534
YOLO-face	0.956	0.588	0.728	0.543
S^3^FD	0.935	0.580	0.716	0.540
SecurePose	0.992	0.990	0.991	0.948

Furthermore, SecurePose exhibited superior precision-recall characteristics and achieved a higher average precision (AP = 0.948) than the other methods assessed (see Table 3, Figs. 5 and 6), suggesting improved face detection performance on clinically recorded gait videos.

Table 4 presents a comparison of computational costs. The YOLO-face was the fastest method, followed closely by Viola-Jones algorithm and SecurePose. It is important to note that the execution settings varied across methods due to implementation constraints, although all computations were performed on the same machine detailed in the system specifications section. Methods marked as ‘NA’ for GPU usage in Table 4 were executed using CPU-only implementations, as GPU acceleration was either unavailable for the provided models or not natively supported in our testing framework. Therefore, the large differences in computational time across methods may partially reflect these implementation differences (e.g., lack of GPU acceleration) rather than intrinsic algorithmic efficiency alone. SecurePose demonstrated efficiency by minimizing CPU, RAM, and GPU resource utilization, though it exhibited slightly higher disk usage compared to other methods. Overall, these results suggest that SecurePose offers a favorable balance between speed and resource consumption relative to the other methods assessed.

**Table 4:** Computational cost of using the methods with the system specifications mentioned in the section II-M.

Methods	Computational time (minutes)	CPU (%)	RAM (GB)	GPU (%)	GPU memory (GB)	Disk SSD (MB/s)
Viola Jones	25	48	0.351	NA	NA	0.1
HOG-based detector	42	9	0.145	NA	NA	0.9
MMOD	7936	10	1.942	NA	NA	0.1
MTCNN	153	8	0.390	26	8.600	0.1
YOLO-face	10.35	8.9	1.2	20	2.00	35
S^3^FD	334	58	0.331	NA	NA	0.1
SecurePose	35	8	0.113	25	0.105	2.5

### Person tracking and identification

While the person tracking and identification strategy implemented in SecurePose likely contributed to higher precision, recall, F1-score, and average precision than existing methods, this methodology had inherent limitations. In 110 of 116 videos (94.8%), the algorithms successfully tracked and assigned unique identifiers (IDs) to all individuals. In 3 videos (2.6%), the unique identifiers of two non-patient individuals were interchanged on 1–2 occasions, attributable to body centroid collisions resulting from close proximity. Algorithm performance was also challenged in these instances by significant occlusion, with the individual in front obscuring > 80% of the other’s body. In the remaining 3 videos (2.6%), the algorithm failed to assign unique identifiers to non-patient individuals due to extensive occlusion. Importantly, these tracking limitations did not affect the face blurring of any individuals in the videos.

### Patient identification

The patient identification algorithm operated under the assumption that the individual positioned closest to the frame’s center would be the primary subject of the video recording. This assumption yielded a 99% success rate (115 of 116 videos) across the dataset. However, in a single instance, the algorithm incorrectly identified a caregiver accompanying the patient as the primary subject.

This rare error highlights the importance of user awareness during implementation of SecurePose. Specifically, added caution should be exercised when applying patient-specific face blurring to videos containing other individuals positioned centrally within the frame (e.g., an assisting caregiver). In such cases, utilizing the “all person blurring” setting within SecurePose is recommended to ensure complete anonymization.

### Improvements after quality check

After automated face blurring, a manual quality check was performed. The manual quality checking achieved ceiling performance with an added time of 48 min for all 116 videos in aggregate (25 s/video). Compared to the time required for ground truth blurring, SecurePose obtained the same performance in less manual effort and time (8.92% of the time taken to blur the videos manually) (Table 5).

**Table 5:** Precision, recall, f1-score, and total time taken by securepose automatic blurring, securepose with manual quality check, and ground truth manual blurring.

Blurring stages	Precision	Recall	F1- score	Time (minutes)
Automated blurring	0.992	0.990	0.991	35
After manual quality check	1.000	1.000	1.000	83
Manual Blurring	NA	NA	NA	930

### OpenPose vs MediaPipe

In our comparison between OpenPose and MediaPipe, we evaluated both algorithms across key performance metrics, including precision, recall, F1-score, and average precision. The results, presented in Table 6, show that OpenPose consistently outperformed MediaPipe across all metrics. However, it is important to note that when integrated into the SecurePose pipeline, MediaPipe achieved higher recall, F1-score, and average precision than all six other existing methods evaluated (see Tables 3 and 6), highlighting its competitive performance in the context of our system despite its lower overall accuracy compared to OpenPose. Table 6 also show a critical trade-off between computational efficiency and pose estimation accuracy for OpenPose and MediaPipe in the context of SecurePose.

**Table 6:** Precision, recall, f1-score and average precision and total time taken for automatic blurring for openpose and mediapipe using securepose.

Methods	Precision	Recall	F1-score	Average precision	Time (minutes)
SecurePose–MediaPipe	0.769	0.952	0.851	0.841	21
SecurePose–OpenPose	0.992	0.990	0.991	0.948	35

### System usability scale (SUS) results

The average SUS score across 10 users was 88.75, indicating a high percentile ranking in usability when compared to other software products assessed using the SUS in the literature^43^ (Table 7).

**Table 7:** System usability scale (SUS) score.

S.No	Questionnaire items	Average of responses	Score contribution
1	I think that I would like to use this software frequently	3.8	2.8
2	I found the software unnecessarily complex	1.9	3.1
3	I thought the software was easy to use	4.8	3.8
4	I think that I would need the support of a technical person to be able to use this software	1.2	3.8
5	I found that various functions in this software were well integrated	4.8	3.8
6	I thought there was too much inconsistency in this software	1.0	4.0
7	I would imagine that most people would learn to use this software very quickly	4.8	3.8
8	I found the software very cumbersome to use	2.2	2.8
9	I felt very confident using the software	4.8	3.8
10	I need to learn a lot of things before I could get going with this software	1.2	3.8
Sum			35.5
Average SUS score = 88.75 (35.5*2.5)			

## Discussion

SecurePose, a novel face blurring algorithm, demonstrates superior performance compared to six existing methodologies when applied to clinically acquired video datasets. SecurePose achieves comparable performance to ground truth manual blurring with a significantly reduced processing time, thus facilitating rapid anonymization for secure sharing of clinically acquired video data.

Quantitative evaluation demonstrates that SecurePose achieves higher precision, recall, F1-score, and average precision for patient face blurring in clinical videos compared to the six existing face detection methods tested. It should be emphasized that SecurePose operates on a fundamentally different detection paradigm (pose-informed detection) compared to the other standalone face detectors evaluated (appearance-based detection). While appearance-based methods rely solely on facial image features, SecurePose leverages structural body information (i.e., the spatial relationship of the face to the neck and shoulders) extracted via pose estimation. This structural context inherently contributes to improved detection performance, particularly in cases of partial facial occlusion or non-frontal head angles, offering a distinct advantage that limits direct comparability with purely appearance-based algorithms. Furthermore, clinical researchers provided a high usability rating for the software. Critically, SecurePose enables the extraction and export of comprehensive full-body kinematic data prior to face blurring, preserving data quality and facilitating subsequent analysis. This automated kinematics extraction capability has the potential to advance clinical assessment and movement research.

Person tracking and patient identification algorithms were used to facilitate accurate and automated patient body kinematics extraction. Notably, SecurePose achieved a 94.8% success rate in the person tracking and a 99% success rate in patient identification. The validation results illustrated that SecurePose can perform person-specific face blurring in the vast majority of clinical gait videos. It is additionally notable that this testing occurred on a clinically-relevant data set using videos recorded on a handheld iPad (thus subject to issues with motion, focus, and illumination). The videos of the patients were recorded during a busy clinic (many of whom had masks or clothing covering body key points, thus complicating kinematics extraction and face identification). This real-world data set makes the high performance of SecurePose even more valuable.

The results of our comparative evaluation between OpenPose and MediaPipe highlight a critical trade-off between computational efficiency and estimation accuracy in the context of SecurePose. While MediaPipe demonstrates superior efficiency and achieves competitive performance in certain metrics, outperforming six existing methods in the SecurePose pipeline, its overall accuracy and robustness under real-world conditions, such as varying lighting and occlusion, are insufficient for reliable clinical deployment. In contrast, OpenPose consistently delivers higher precision and more stable keypoint detection, which is essential for accurate tracking and effective blurring. These findings underscore that the choice of pose estimator significantly impacts the system’s performance and reliability, indicating that the robustness of SecurePose relies not only on its tracking and blurring logic but also on the quality of the underlying pose estimation module.

### Limitations and future work

While SecurePose was validated for face blurring and body kinematics extraction on a clinically relevant gait examination, future work should extend validation to other clinically relevant video-based assessments, such as seated or recumbent tasks and other exam maneuvers. Although the primary objective of this study was to develop reliable software for clinical applications, the algorithms were not optimized for computational time. Future iterations will incorporate multi-threading and optimized algorithms to reduce automated kinematics extraction and face blurring processing times.

To address different recording needs, SecurePose offers two distinct blurring modalities: 'patient-only’ and 'all-people.' The 'patient-only’ mode utilizes a specific patient identification algorithm which employs a rule-based approach assuming the patient’s presence for > 80% of the video duration and proximity to the center. This approach is specifically optimized for standardized video-based physical examinations, such as gait analysis, hand open-close tasks, and similar movement disorder assessments, where the patient is the primary subject and remains centrally positioned. Since the majority of clinical tasks adhere to these recording protocols, the centroid assumption is largely valid for standard medical use. However, variations in camera angle or distance that displace the patient to the periphery constitute a limitation for the 'patient-only’ mode, as the algorithm strictly presumes that the patient remains centrally positioned. This approach successfully ‘patient-only’ blurred in 99% of gait videos used in this study. Distinct from this specific clinical mode, the software provides an 'all-people’ blurring feature. This mode operates independently of the patient identification logic and is capable of blurring all faces in the video. It is therefore effective for tasks involving multiple people or recordings where subjects do not remain in the center. Future work will focus on generalizing the specific patient identification logic to further handle scenarios where the patient moves peripherally without requiring the ‘all-people’ setting.

Furthermore, it is important to acknowledge that SecurePose was developed and validated exclusively on gait videos of children with cerebral palsy recorded in a specific clinical setting. While the pipeline is designed as a general solution for clinical video anonymization and kinematics extraction, its generalizability and performance on other clinical tasks (e.g., seated examinations, upper limb tasks), different age groups, varying recording devices, camera positions, or extreme lighting conditions remain untested and warrant further investigation.

Currently, the software operates solely on a local server, ensuring patient data privacy but requiring substantial computational resources. A web-based application leveraging cloud computing would improve accessibility across institutions and facilitate automated kinematics extraction and face blurring.

## Conclusion

This paper presents the development and validation of open-source software called SecurePose for automated face blurring and human kinematics extraction from clinically recorded gait videos. SecurePose achieved a precision of 0.992, recall of 0.990, F1-score of 0.991, and average precision of 0.948 for automated face detection in a real-world clinical dataset of 116 gait videos of individuals with cerebral palsy. Furthermore, SecurePose achieved face blurring performance comparable to manual blurring in 8.92% of the time. Usability testing confirmed the software’s user-friendliness, as measured by the System Usability Scale. These findings demonstrate SecurePose’s efficacy, efficiency, and usability for face blurring and kinematics extraction in clinical gait videos.

## Data Availability

The software is freely available on https://sourceforge.net/projects/securepose/.
